# A Programmable and Portable Electromagnetic Microfluidic Platform for Droplet Manipulation

**DOI:** 10.3390/bios16040196

**Published:** 2026-03-31

**Authors:** Chaoze Xue, Shilun Feng, Wenshuai Wu, Zhe Zhang, Jianlong Zhao, Gaozhe Cai, Ting Zhou

**Affiliations:** 1 School of Microelectronics, Shanghai University, Shanghai 201899, China; 23723685@shu.edu.cn (C.X.); zhangzhe23@shu.edu.cn (Z.Z.); jlzhao@mail.sim.ac.cn (J.Z.); 2 State Key Laboratory of Transducer Technology, Shanghai Institute of Microsystem and Information Technology, Chinese Academy of Sciences, Shanghai 201899, China; shilun.feng@mail.sim.ac.cn; 3 Key Laboratory of Environmental Medicine and Engineering, Ministry of Education, Department of Nutrition and Food Hygiene, School of Public Health, Southeast University, Nanjing 210009, China; 101013696@seu.edu.cn; 4 Shanghai Frontier Innovation Research Institute, Shanghai 201108, China

**Keywords:** droplet manipulation, magnetic digital microfluidics, programmable, portable, electromagnetic control system

## Abstract

Droplet manipulation constitutes a fundamental operation in numerous bio-microfluidic applications, including but not limited to medical diagnostics and targeted drug delivery. Among the various technologies developed for this purpose, magnetic digital microfluidics (MDMF) has emerged as a compelling approach due to its inherent advantages of contamination-free actuation, low cost, and configurational flexibility. Nevertheless, conventional MDMF remains constrained by its reliance on bulky instrumentation and substantial power consumption for generating controllable magnetic fields, which limit its in-field applications. To address these limitations, this work presents a programmable and portable electromagnetic microfluidic droplet manipulation platform that synergistically integrates static and dynamic magnetic fields to enable non-contact, high-precision droplet control under ultra-low power conditions. The proposed system comprises an electromagnetic actuation module, a permanent magnet, and a glass substrate coated with Teflon film. The entire system is secured by a PMMA support structure, within which a glass substrate is mounted and spatially separated from the permanent magnet. The PMMA support is fabricated using a milling process, offering a simple manufacturing procedure and high structural reusability and reproducibility. The control logic is implemented on a field-programmable gate array (FPGA) development board, facilitating fully autonomous operation powered by a standard battery. The platform operates at a low voltage of 3.5 V and a driving current of 180 mA, corresponding to a total power consumption of merely 0.63 W, while achieving robust manipulation of droplets in the volume range of 0.5 to 5 μL. A maximum average droplet velocity of up to 0.6 cm/s was attained under optimal conditions. The proposed platform offers a scalable and energy-efficient solution for portable droplet-based assays and holds significant promise for integration into point-of-care diagnostic tools and field-ready biochemical analysis systems. The platform demonstrates excellent operational stability and reproducibility, as validated by repeated actuation experiments with a positioning deviation of approximately 0.1 mm under optimized conditions. The fabrication process also exhibits high reliability with consistent performance across multiple experimental runs.

## 1. Introduction

Conventional bioanalytical and chemical assays typically rely on manual pipetting for solution transfer. However, such approaches are prone to human error and risk cross-contamination, thereby compromising experimental accuracy and reliability. With the advancement of microfluidic technologies, continuous-flow microfluidics has increasingly supplanted manual operations in biological applications due to its high throughput, operational stability, and precise fluid handling capabilities [1,2,3,4]. Nevertheless, the confined architecture of microchannels inherently restricts flexible and programmable droplet manipulation, while its reliance on external pressure sources or pump-based flow control may also limit its in-field applications [5]. Digital microfluidic (DMF) technologies have emerged as a promising solution to overcome the inherent limitations of continuous-flow systems. By enabling discrete and programmable control over individual droplets on open-surface, DMF allows for a wide range of operations, including droplet transport, merging, splitting, and on-chip reactions with high flexibility, minimal risk of contamination, and low sample consumption [6,7,8]. Currently, DMF primarily relies on acoustic [9,10,11,12,13], optical [14,15], thermal [16], electric fields [17,18] and magnetic fields [19,20,21,22] to manipulate droplets with high precision and responsiveness.

Electrowetting-on-dielectric (EWOD), a representative electric-field-driven DMF approach, operates by the electrostatic force acting on the surface of the droplet across patterned electrodes beneath a dielectric layer [23]. This mechanism enables highly programmable and flexible transport of discrete droplets on open surfaces and is well-suited for small-volume reaction systems [24,25,26,27,28,29]. However, most EWOD-based platforms require direct contact between the droplet and the chip’s dielectric and hydrophobic layers. Prolonged operation often leads to surface contamination and degradation of the hydrophobic coating, adversely affecting device performance and reliability. Moreover, many organic solvents tend to lose their functional integrity under high electric fields and may even corrode the electrode material, thereby limiting the system’s chemical compatibility and operational lifespan [30,31,32].

Magnetic microfluidics has emerged as an attractive alternative for droplet actuation in digital microfluidic systems, offering key advantages such as non-contact manipulation and low driving voltages. In contrast to EWOD-based platforms, where droplet trajectories are confined to pre-patterned electrode grids, magnetic approaches enable programmable and unconstrained transport via reconfigurable magnetic fields or magnetically functionalized substrates, providing greater adaptability for complex fluidic operations. Manipulation of droplets that incorporate or are interfaced with magnetic particles is typically achieved by applying external magnetic fields generated by either permanent magnets [33,34,35] or electromagnets [36,37,38,39,40], allowing for precise spatial and temporal control across a broad range of chemical and biological applications.

Several magnetically actuated droplet manipulation strategies have been developed to date. Based on the different methods of controlling the properties of the droplets, it can mainly be categorized into invasive control and non-invasive control. The former mainly involves magnetic droplet manipulation based on magnetic elements contained within the droplets, including static magnetic field drive generated by permanent magnets [22,41] and electromagnetic field generation through integrated micro-magnetic coils [21,42]; while the latter includes magnetic droplet manipulation based on magnetic fluids [40,43] and droplet magnetic field manipulation based on magnetic superhydrophobic surfaces [44,45]. This research mainly focuses on the improvement and innovation of the invasive magnetic drive method. Among these kinds of methods, systems employing bi-axial motorized stages to reposition permanent magnets beneath the chip surface offer stable and strong actuation forces with a high degree of programmability [46]. However, such static-field-based approaches typically involve bulky instrumentation and substantial power consumption, limiting their integration into portable or point-of-care platforms. To address these limitations, Katsikis et al. introduced soft magnetic tracks patterned with permalloy on the chip surface to generate localized magnetic gradients [47]. While this design significantly reduces system size, it frequently encounters trajectory distortion and operational bottlenecks, which compromise both accuracy and scalability. Other efforts have focused on electromagnetic actuation strategies. For instance, spatially addressable arrays of electromagnetic rods have been employed to generate localized fields, offering sufficient actuation strength but at the cost of high-power consumption dissipation and undesired thermal effects, which may compromise sensitive biochemical assays [42,48]. Lehmann et al. further advanced this concept using on-chip integrated microcoils to enable transport, mixing, and bead-based separation in magnetically functionalized droplets [42]. However, these planar coils are typically limited in current handling capacity, producing insufficient magnetic force to mobilize entire droplets and instead displacing only magnetic nanoparticles within them. Collectively, while static magnetic systems offer strong actuation at the cost of system size and mobility, electromagnetic systems favor miniaturization and programmability, yet often suffer from limited force output and thermal constraints. These trade-offs underscore the need for a hybrid magnetic actuation strategy that leverages the strengths of both static and dynamic magnetic fields for robust, precise, and energy-efficient droplet manipulation.

An often overlooked aspect is that programmability not only reflects the flexibility of droplet manipulation; more importantly, it enables application-specific customization without altering the underlying electromagnetic control system architecture. For example, beyond simple droplet transport, oscillatory droplet motion can be achieved by modifying the programming logic. In addition, the driving scheme can be dynamically adapted to accommodate different upper-layer microfluidic configurations. Notably, all these functionalities are realized without redesigning the coil array, thereby significantly enhancing the reusability and adaptability of the electromagnetic control platform hardware.

**Figure 1:**
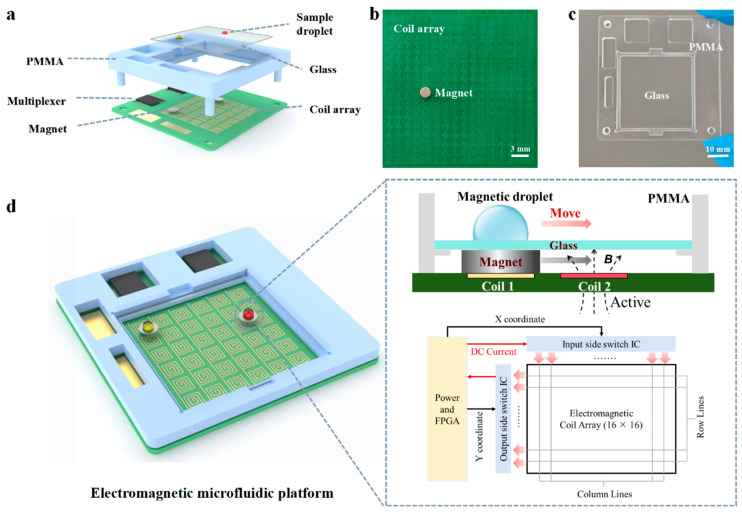
Schematic illustration of the droplet manipulation platform. (**a**) Schematic illustration of the electromagnetic microfluidic platform, showing all major functional components. The system consists of a multilayer architecture including the printed circuit board (PCB) within a coil array (bottom), the cylindrical permanent magnets (middle), and the glass substrate (top). (**b**) Picture of the planar microcoil array. (**c**) Picture of the PMMA support mounted with a hydrophobic glass substrate. (**d**) Schematic overview of the electromagnetic control system, illustrating the structural framework of the PCB layout and the workflow of droplet manipulation.

In this work, we present a programmable and portable electromagnetic microfluidic droplet manipulation platform capable of flexible manipulation of magnetically responsive microdroplets, including transportation and merging. This proposed microfluidic system comprises an electromagnetic actuation module, a permanent magnet, and a glass substrate coated with Teflon film. As shown in Figure 1, the electromagnetic actuation module integrates a 16 × 16 array of microfabricated square coils (1.5 mm × 1.5 mm) for driving N52 permanent magnets (2.5 mm diameter), a field-programmable gate array (FPGA) based control unit, and a poly (methyl methacrylate) (PMMA) support for mounting the glass substrate. This configuration enables low-voltage, low-current actuation, significantly reducing system power requirements and enhancing portability.

## 2. Materials and Methods

### 2.1. Materials

N52 permanent magnet was purchased from Xinyongquan Industrial Co., Ltd. (Guangdong, China). Teflon AF 2400 solution was purchased from Chemours Chemical (Shanghai) Co., Ltd. (Shanghai, China) and used as the hydrophobic coating reagent. The deionized water was prepared by the ultrapure water system Arium^®^ Mini Plus (Gottingen, Germany). The mineral oil was purchased from Sigma-Aldrich (St Louis, MO, USA). The water-based ferrofluid EMG 900 was purchased from Ferro Tec Co., Ltd. (Cleveland, 566 Exchange Court, Livermore, CA, USA). The layout of the PCB was designed and implemented by the JLCEDA software (Version 6.5.51) of Shenzhen JLC Technology Group Co., Ltd. (Guangdong, China). The production of the PCB was carried out by Shenzhen Nanbang Innovation Co., Ltd. (Guangdong, China).

### 2.2. The Design of the Electromagnetic Microfluidic Platform

As shown in Figure 1a, the proposed programmable and portable electromagnetic droplet manipulation platform comprises two primary components: an electromagnetic control system and a PMMA support mounted with a glass substrate. Distinct from conventional magnetic droplet manipulation systems, the electromagnetic module in this work integrates a microcoil array with millimeter-scale cylindrical N52 permanent magnets. The core mechanism relies on selectively activating microcoils embedded on the PCB to generate localized magnetic field gradients that drive the motion of the magnets. As illustrated in Figure 1b, the permanent magnets can be flexibly actuated by the microcoil array patterned on the PCB. Each square microcoil has a lateral dimension of 1.5 mm, while the permanent magnet used has a diameter of 2.5 mm and a thickness of 1 mm. The thickness of the copper wire is 1 oz, and the width of the wire is 0.1 mm. The center-to-center spacing between adjacent coils is 1.75 mm (only a partial array was shown in Figure 1). Each coil is wound three turns counterclockwise from the input end to the center with a wire spacing of 0.25 mm. The transparent PMMA support was fabricated by micromilling and used for mounting the glass substrate (Figure 1c). Both materials were selected for their high optical transparency, facilitating direct visualization of the spatial relationship between droplets, magnets, and underlying coils.

The fabrication tolerances of the PCB-based microcoil array were controlled within typical industrial PCB fabrication tolerances, including a trace width variation of approximately ±10 μm, coil geometry deviation within ±25–50 μm, and layer-to-layer alignment accuracy within ±50 μm. Such dimensional precision ensures minimal variation in magnetic field distribution and contributes to consistent and reliable actuation performance.

The working principle of droplet actuation is depicted in Figure 1d. When adjacent coils are sequentially energized, a localized magnetic field is generated around each active coil in accordance with Ampère’s law. Once the magnetic field strength surpasses a threshold, the magnet is attracted toward the energized coil. Acting as a magnetic force amplifier, the magnet enhances both the magnitude and precision of the magnetic field applied to the overlying droplet, enabling accurate and contactless droplet manipulation within the microfluidic chip. In the illustrative example, the first coil remains de-energized and thus generates no magnetic field, while the second coil is activated and produces a localized magnetic field. According to the right-hand rule, the direction of the induced magnetic field can be determined, driving the magnet from its initial position to the target position. By programming the multiplexer to switch coil activation in a predefined sequence, the magnet can be guided along a custom trajectory, thereby transporting the magnetic droplet in a controlled manner.

### 2.3. Composition and Principle of the Electromagnetic Control System

**Figure 2:**
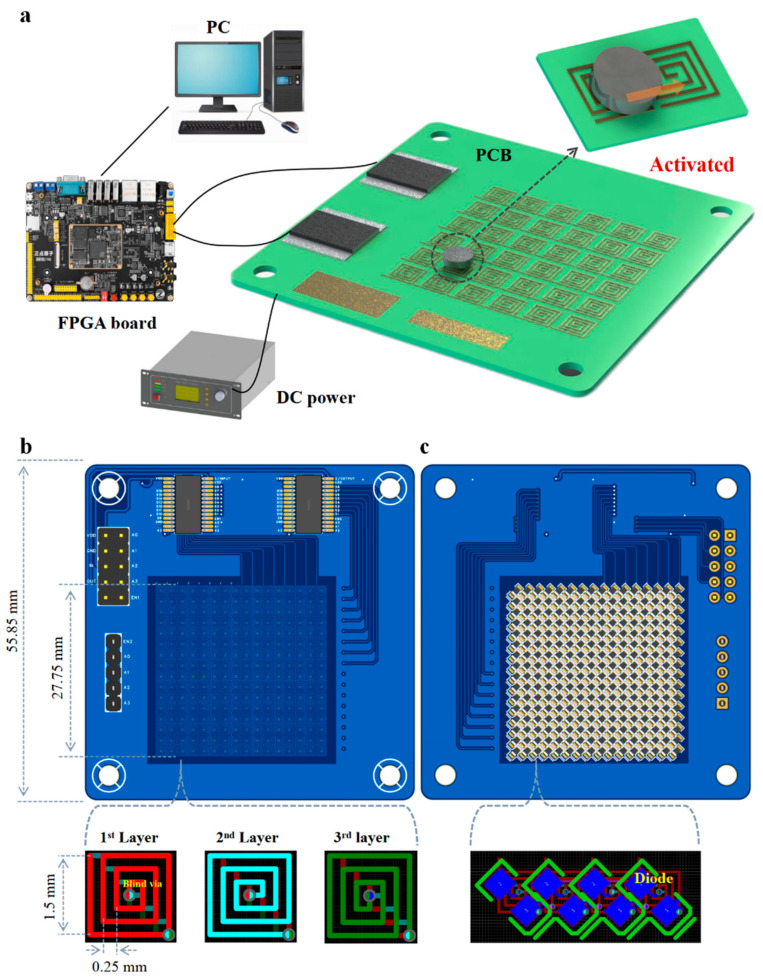
Hardware connection diagram of the electromagnetic control system and design of PCB layout. (**a**) Schematic illustration of the electromagnetic control system. (**b**) Schematic illustration of the top-layer PCB layout. Interlayer connectivity between stacked coils is achieved via blind vias. (**c**) Schematic illustration of the bottom-layer PCB layout. Each coil is connected in series with a discrete diode positioned directly beneath it, forming a 1:1 coil-to-diode configuration.

The electromagnetic control system developed in this study comprises a multilayer PCB, N52 permanent magnets, an FPGA control module, and a regulated DC power supply. The actuation circuitry integrates an array of planar microcoils, addressable via a pair of ADG1606 analog multiplexers, alongside protective diodes and dual-header interfaces for external communication and power delivery. As illustrated in Figure 2a, a control program preloaded on the host computer is transmitted to the FPGA development board, which subsequently communicates with the multiplexer on the PCB. This enables programmable activation of the microcoil array in a user-defined sequence, thereby guiding the permanent magnet to move stepwise along the corresponding energized coils. In this manner, precise and reconfigurable magnetic control can be achieved. The multiplexer is powered directly by the FPGA board, operating at a supply voltage of 5 V, while the conduction current for the microcoils is sourced from an external DC power supply to ensure sufficient actuation force.

The layout of the PCB was shown in Figure 2b,c with the final board dimensions constrained to 55.85 mm × 55.85 mm. To optimize spatial efficiency while maintaining high-resolution magnetic actuation, a 16 × 16 coil matrix was designed at the core of the PCB, occupying 27.75 mm × 27.75 mm. Each coil features a square geometry with a 1.5 mm edge length and comprises three concentric turns to enhance magnetic field intensity. The coils were fabricated with a spacing of 0.25 mm, and all interconnect traces were routed with a minimum width of 1 mm to support stable current delivery under continuous operation. To enable programmable activation of specific coils, two ADG1606 multiplexers were employed to selectively address rows and columns within the array. Control signals were generated from an FPGA module, which can execute precompiled instructions transmitted from a host computer, allowing dynamic and flexible coil switching sequences. Power routing was managed through two onboard pin headers (5-pin and 10-pin), which interfaced with the FPGA and DC supply, respectively. The multiplexers were operated at a voltage of 5 V, which was supplied by the FPGA board. The coil currents were sourced from the external DC power unit to ensure sufficient actuation force. Each coil was wound in a counterclockwise configuration to produce an upward-oriented magnetic field. Vertical interlayer connectivity was established through blind and buried vias, each with an inner diameter exceeding 0.25 mm to ensure reliable electrical continuity and mitigate short-circuit risks. To prevent unintended mutual interference between adjacent coils, particularly relevant in high-density layouts, each coil was serially connected with a diode on the bottom layer of the PCB. This diode-coil cascading topology, illustrated in Figure 2c, was used to ensure unidirectional current flow and suppress crosstalk during rapid switching events. This structural and electrical configuration provides a compact yet functionally robust electromagnetic control platform capable of executing precise, low-power, and reconfigurable droplet manipulation protocols in resource-limited or portable diagnostic settings.

The control architecture of the proposed platform was built upon an FPGA. The desired logic configuration is synthesized and compiled into a bitstream, which is then downloaded onto the FPGA to implement the hardware logic in situ. Unlike conventional microcontrollers or platforms based on sequential execution paradigms, such as C/C++-based processors or Arduino systems, FPGAs inherently support true hardware-level parallelism. Each logic block can operate independently and concurrently, allowing the simultaneous execution of hundreds or even thousands of control tasks. This parallel processing capability makes the FPGA particularly well-suited for scenarios requiring high-throughput and time-synchronized operations, such as the concurrent control of multiple magnetic elements within the microcoil array.

### 2.4. Surface Treatment

The droplet motion within the microfluidic system is primarily governed by the balance between magnetic driving force and surface friction. To enhance actuation performance and reduce resistive forces, the glass substrate surface was modified to become hydrophobic. In this work, a square glass substrate measuring 34 mm × 34 mm was employed. Prior to surface treatment, the substrate was thoroughly cleaned with 75% ethanol to eliminate surface contaminants and ensure uniform coating quality. A hydrophobic layer was then applied by spin-coating 5 μL of Teflon solution onto the glass surface, followed by thermal curing on a hot plate for a defined period. After curing, the substrate was cooled to ambient temperature prior to use. This treatment significantly reduced the frictional resistance encountered by the droplet during motion, enabling smoother, more efficient magnetic actuation across the chip surface.

In addition, the surface treatment process, including Teflon coating and thermal curing, was performed under controlled conditions to minimize variations in surface wettability. Experimental observations confirmed that the fabricated devices exhibited consistent droplet manipulation behavior across multiple samples and repeated trials, demonstrating good reproducibility and reliability of the fabrication process.

## 3. Results and Discussion

### 3.1. Magnetic Simulation of the Electromagnetic Coils

The core operating principle of the electromagnetic control platform is grounded in Ampère’s law, in which magnetic fields generated by current-carrying coils actuate permanent magnets, which in turn drive droplet motion. Consequently, both the intensity and uniformity of the magnetic field generated by the coils play a critical role in determining the precision and robustness of droplet manipulation. To quantitatively evaluate magnetic field intensity and uniformity across different coil configurations, finite element simulations were conducted in COMSOL Multiphysics (Version 2020). Three distinct coil structures—comprising one, two, and three layers of coils—were analyzed to assess the influence of vertical stacking on magnetic field superposition. However, magnetic field superposition must overcome magnetic field attenuation. The magnetic field generated by a planar coil, which decays with increasing vertical distance, can be approximated as that of a permanent magnet or a magnetic dipole. The specific formula is as follows:

(1) $$B=\frac{\mu_{0}}{4\pi }\cdot \frac{m}{r^{3}}$$

where B is the magnetic field intensity, $\mu_{0}$ is the vacuum permeability, approximately $4\pi \times 10^{-7}\;H/m$, m is the magnetic dipole moment, r is the attenuation distance. It is evident that the magnetic field intensity B decays proportionally to the cube of the distance r. The specific formula is as follows:

(2) $$B\propto \frac{1}{r^{3}}$$

Given that the cumulative magnetic field in multilayered configurations is sensitive to interlayer spacing, a vertical spacing of 150 μm was adopted in the simulation to minimize field attenuation. The driving current was uniformly set to 180 mA for all cases.

**Figure 3:**
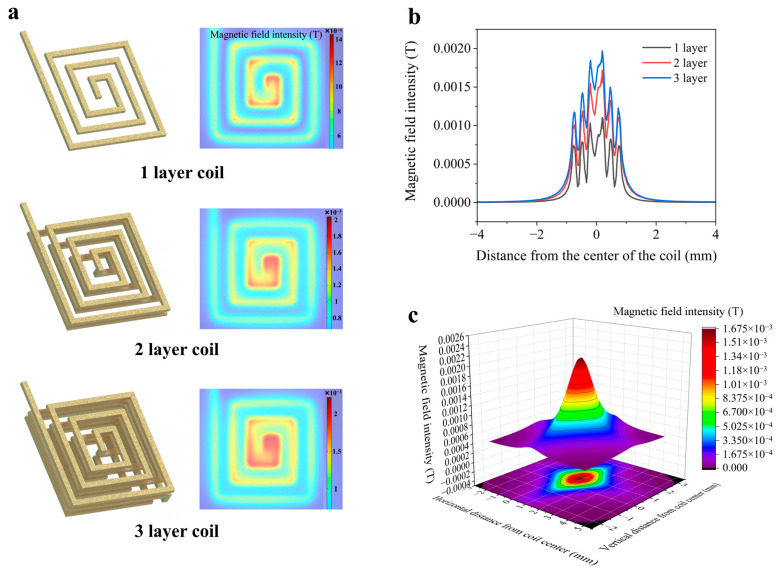
The simulation results of the electromagnetic coil. (**a**) Structural models and COMSOL simulation results for single-layer, double-layer, and triple-layer planar coil configurations designed in this study. Each model was simulated under identical excitation conditions (180 mA current) to evaluate magnetic field intensity and uniformity. (**b**) Simulated magnetic field distribution for single-layer, double-layer, and triple-layer coil structures at a horizontal plane located 25 μm above the coil surface. (**c**) Three-dimensional distribution of magnetic field intensity generated by the triple-layer coil configuration.

As shown in Figure 3a, the three-layer coil configuration exhibited the most uniform magnetic field distribution in the central region of the coil surface. To further assess the effective field intensity at the top surface of the coil, accounting for the presence of an insulating solder mask layer typically deposited during PCB fabrication, a horizontal plane located 25 μm above the top coil layer was introduced in the simulation. The resulting magnetic field intensity, illustrated in Figure 3b, demonstrated that the three-layer configuration significantly outperformed its one-layer and two-layer counterparts, achieving a peak magnetic flux density of 1.97 × 10^−3^ T. This enhanced magnetic field intensity enables reliable magnet actuation with lower current input, thus improving energy efficiency. To visualize the spatial magnetic field distribution in greater detail, a three-dimensional surface plot was generated based on the three-layer configuration, as shown in Figure 3c. The x- and y-axes represent lateral positions across the coil plane. Meanwhile, the z-axis indicates magnetic field intensity, with the coordinate origin located at the coil center.

Based on these simulation results, the three-layer coil structure was selected as the magnetic field source for the final electromagnetic actuation module, owing to its superior field intensity and spatial uniformity.

### 3.2. Stability of the Electromagnetic Control System

**Figure 4:**
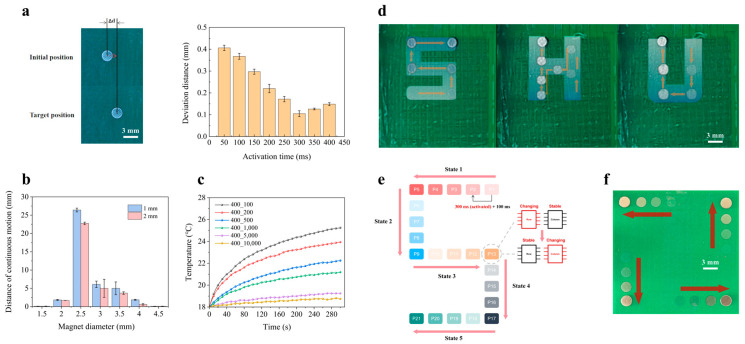
Experimental evaluation of the stability of the electromagnetic control system. (**a**) Positional accuracy of magnetic manipulation under different coil activation durations, ranging from 50 ms to 400 ms. (**b**) Effect of magnet diameter and thickness on the maximum single-step driving distance achieved by the electromagnetic control system. (**c**) Effect of coil activation interval on the surface temperature of the microfluidic chip (2.7 mm from the surface of microcoils). (**d**) Successfully manipulating the permanent magnet along three representative trajectories shaped as the letters “S,” “H,” and “U.” (**e**) Magnetic manipulation along an “S”-shaped trajectory using a 400 ms cycle (300 ms activation and 100 ms interval). 90° directional turns were achieved through real-time reconfiguration of the row/column addressing scheme via a state machine implemented on the FPGA controller. (**f**) Demonstration of simultaneous manipulation of four magnets within a 16 × 16 microcoil array.

The accuracy with which the permanent magnet can be positioned forms the operational basis of magnetic droplet manipulation. Given the spatial confinement of the magnetic field generated by each microcoil, excessive displacement from the coil center during magnet translation may result in the loss of magnetic coupling, thereby impairing the continuity of subsequent actuation steps. Ensuring the magnet resides as close as possible to the center of the target coil is thus imperative for reliable operation. To evaluate the relationship between coil activation duration time and the accuracy of magnet manipulation, deviation which refers to the discrepancy between the actual displacement of the magnet’s center after a single-step actuation (∆d) and the theoretical center-to-center distance between the initial and target coils was defined to quantify the accuracy of magnet manipulation and systematic experimental evaluations were performed across eight discrete activation time dose ranging from 50 ms to 400 ms. As illustrated in Figure 4a, the positional deviation of the magnet exhibited a monotonic decrease as activation time increased from 50 ms to 300 ms (Excel S2). This is attributed to the longer magnetic field exposure, which facilitates sufficient time for the magnet to migrate toward the point of maximal field intensity. Beyond 300 ms, a marginal degradation in accuracy was observed, likely due to micro-oscillations induced by prolonged exposure to electromagnetic field gradients. An activation duration of 300 ms was found to yield the highest positional fidelity, with an average center-to-center deviation between the magnet and the activated coil of approximately 0.1 mm. The observed positioning deviation can be attributed to several sources of measurement error. These include fabrication-induced tolerances in coil geometry and alignment, slight misalignment between the permanent magnet and the coil center during actuation, and transient variations in the magnetic field during rapid switching. In addition, surface inhomogeneity and friction variations on the hydrophobic substrate may also contribute to minor deviations in motion. Based on the experimental results, the positioning accuracy of the system can be quantified by the average deviation between the actual and expected magnet positions, which was measured to be approximately 0.1 mm under optimized conditions. This level of accuracy is sufficient for most droplet-based microfluidic applications, which was therefore adopted as the standard actuation duration for all subsequent experiments.

In addition to the activation time of the coil, the geometric dimensions of the magnet can significantly influence translational performance due to the spatial matching between the permanent magnet and the magnetic field distribution induced by the energized coil. To investigate the correlation between the distance of continuous motion of the magnet and its geometric dimensions, magnets of varying diameters and thicknesses were evaluated under identical driving conditions. To ensure the reliability and consistency of the experimental results, each magnet with a different geometric configuration was tested under identical actuation conditions, including a fixed driving current of 180 mA, an activation duration of 300 ms followed by a 100 ms idle interval, and a uniform lateral movement trajectory. As shown in Figure 4b, magnets with diameters of 1.5 mm failed to respond to actuation, primarily due to insufficient surface area for effective magnetic coupling. In contrast, 4.5 mm magnets, though better aligned with the field, were found to be prohibitively heavy, leading to excessive frictional resistance. Among the tested configurations, a magnet with a diameter of 2.5 mm and a thickness of 1 mm exhibited optimal actuation performance, demonstrating consistent translational motion across the full extent of the coil array, achieving a maximum displacement of up to 26.25 mm, thereby fully leveraging the spatial capacity of the system. Based on the previously optimized actuation timing parameters, the magnet could be reliably actuated at a velocity of up to 0.6 cm/s. This geometry thus represents a critical balance between sufficient magnetic responsiveness and manageable mechanical resistance.

Thermal management is a critical consideration in bioanalytical systems, particularly in applications involving thermally labile reagents, enzymes, or cellular components. To assess the thermal stability of the proposed platform under operational stress, an accelerated heating scenario was designed to represent a worst-case thermal condition. In this test, a single coil was repeatedly energized for 300 ms while varying the actuation intervals (100 ms, 200 ms, 500 ms, 1000 ms, 5000 ms, and 10,000 ms) over a continuous 5-min period. The temperature at a point located 2.7 mm above the coil center was monitored in real time using a temperature sensor (DS18B20), which is mounted on the glass surface using hot-melt adhesive. Its output terminal was connected to the C51 development board to display temperature variations in real time. As illustrated in Figure 4c, a clear inverse correlation was observed between the actuation interval and the temperature rise rate. Nevertheless, the peak temperature under the most thermally aggressive condition (100 ms interval) did not exceed 26 °C, which remains well below the critical thresholds known to affect most biological and biochemical assays.

The favorable thermal characteristics of the platform are attributed to its inherently low-power operation, characterized by a voltage of 3.5 V and a driving current of 180 mA, yielding a total power dissipation of approximately 0.63 W. Moreover, the temporally discrete and spatially localized activation pattern of the microcoil array during standard droplet manipulation routines further minimizes cumulative thermal load. These characteristics underscore the platform’s compatibility with thermosensitive biochemical processes and reinforce its applicability in temperature-constrained settings, such as point-of-care diagnostics and in situ bioanalytical assays.

### 3.3. Magnet Manipulation

To verify the platform’s capability for continuous and multi-directional magnetic actuation, including both bidirectional (back-and-forth) translation and orthogonal (90°) turning, a series of predefined trajectories was implemented.

In this study, only one control chip was employed to address and activate the coil array, so the advantages of FPGAs were not fully exploited. However, for larger-scale coil arrays, more control chips are required to drive the circuitry. A single microcontroller has a limited capacity to drive chips and executes code sequentially. In contrast, an FPGA implements control logic via hardware circuits, whose parallel architecture enables simultaneous operation of all control chips. Moreover, FPGAs exhibit shorter clock-cycle delays, allowing multiple magnets to be actuated synchronously. For complex biological detection procedures, this can significantly reduce the overall processing time.

Corresponding control sequences were compiled and uploaded to the FPGA to drive the multiplexer array. The coil matrix was controlled by an FPGA-based driving system employing a finite state machine (FSM) architecture for addressing both row and column multiplexers. As illustrated in Figure 4e, the “S”-shaped path comprises five discrete FSM states. Within each state, the actuation process consists of four timing steps, each with a 400 ms duration (comprising a 300 ms activation window and a 100 ms idle interval). Certain positions along the trajectory, such as P5, P13, and P17, require dynamic switching between row-wise and column-wise addressing modes to facilitate directional changes. Taking position P13 as an example, the FSM logic transitions the row multiplexer from a continuously changing signal mode to a static signal state, while concurrently switching the column multiplexer from a fixed-state mode to a time-varying signal input. This transition enables the magnet to execute a 90° turn with high fidelity, driven purely by coil activation sequences without external intervention.

During the experiments, the driving current was fixed at 180 mA, with a coil activation time of 300 ms and an inter-pulse interval of 100 ms. As shown in Figure 4d and Figure S1. Movie, the permanent magnet was successfully actuated along three representative trajectories shaped as the letters “S”, “H”, and “U”. The magnet followed these paths smoothly and without deviation, demonstrating that the coil activation strategy ensures robust magnetic coupling during consecutive transitions. The results confirm that at each time step, the activated magnetic field of coils remains sufficiently overlapped with those of its immediate neighbors (up, down, left, and right), thereby achieving seamless, dead-zone-free magnet mobility across complex paths.

Notably, the demonstrated “S”, “H”, and “U” trajectories are not merely predefined paths but serve as representative examples of programmable droplet manipulation. These complex motion patterns require coordinated switching between row and column addressing modes, which is achieved through the FPGA-based FSM control.

This programmability enables the execution of multi-step and multi-directional droplet operations, demonstrating the capability of the system to perform complex fluidic tasks beyond simple linear transport. Such flexibility is essential for applications involving automated assay workflows and programmable biochemical processes.

Furthermore, the system’s scalability was demonstrated by simultaneously actuating up to four individual magnets, as depicted in Figure 4f. Each magnet was independently guided along a programmed path without interference, validating the platform’s capacity for parallel, programmable, and spatially decoupled actuation—an essential feature for future applications in multiplexed droplet operations.

### 3.4. Surface Treatment

**Figure 5:**
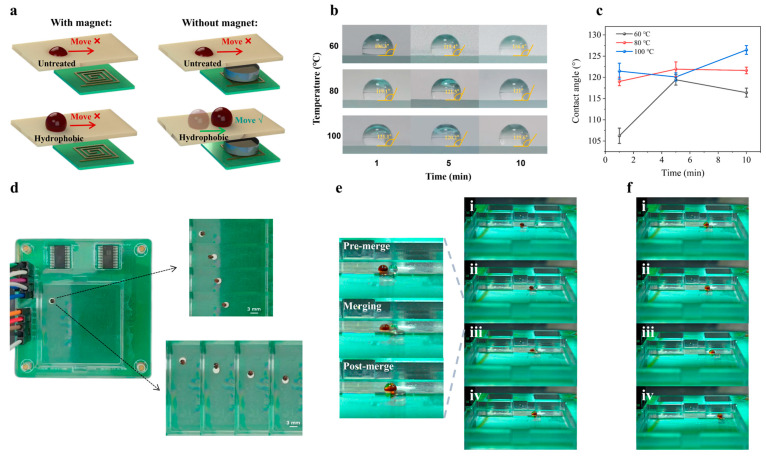
Surface treatment and droplet manipulation. (**a**) Comparison of droplet magnetic manipulation methods. (**b**,**c**) Optical photograph showing the effect of heating temperature and duration on the contact angle after Teflon-based hydrophobic surface treatment. (**d**) Demonstration of smooth horizontal and vertical droplet transport on the electromagnetic microfluidic platform under optimized actuation parameters (180 mA, 300 ms activation, 100 ms interval). (**e**) Optical photograph of the manipulation of the merging between a magnetic and a non-magnetic droplet (each 2.5 μL). (**f**) Optical photograph of manipulating the post-merging magnetic droplet (5 μL).

Droplet manipulation under magnetic actuation, whether in the presence or absence of permanent magnets, is fundamentally inhibited on untreated surfaces lacking hydrophobic modification, as demonstrated in Figure 5a. This phenomenon is primarily attributed to the substantial resistive forces existing at the droplet–substrate interface. Consequently, to facilitate efficient and smooth droplet manipulation within microfluidic platforms, surface treatment imparting hydrophobicity is indispensable.

On a hydrophobic substrate, magnetic droplets are subjected mainly to two resistive forces: the frictional force $F_{f}$, arising from the droplet–substrate interaction, and the adhesion force $F_{adhesion}$ induced by contact angle hysteresis. Successful magnetic actuation necessitates that the driving magnetic force $F_{d}$ surpass both $F_{f}$ and $F_{adhesion}$. The frictional force can be quantitatively described as follows:

(3) $$F_{f}=k\left(F_{my}+mg\right)$$

where k denotes the coefficient of friction between the droplet and substrate, m is the droplet mass, g is the gravitational acceleration, and $F_{my}$ represents the vertical component of the magnetic force. This relationship underscores the imperative to minimize both the friction coefficient and droplet mass to reduce frictional resistance. The adhesion force $F_{adhesion}$ resulting from contact angle hysteresis is expressed as follows:

(4) $$F_{adhesion}=\pi \cdot \gamma \cdot D\cdot \left({cos\theta }_{R}-{cos\theta }_{A}\right)$$

where γ (N/m) signifies the interfacial tension between liquid and air, D is the droplet’s basal diameter, $\theta_{A}$ and $\theta_{R}$ represent the advancing and receding contact angles, respectively. Reduced contact angle hysteresis, defined by $\theta_{A}-\theta_{R}$, facilitates droplet manipulation. Notably, when $\theta_{A}$ and $\theta_{R}$ approach 180°, the adhesion force becomes negligible, thus rendering $F_{adhesion}$ a minor factor in droplet displacement. Therefore, maximizing the contact angle is critically important for enhancing droplet transport efficiency. The substantial enhancement of contact angle achievable through hydrophobic treatment is theoretically described by the Cassie–Baxter model:

(5) $${cos\theta }^{*}=f_{s}cos\theta +f_{v}cos\theta_{v}$$

$\theta^{*}$ denotes the apparent contact angle post-treatment, θ is the intrinsic contact angle prior to treatment, $f_{s}$ and $f_{v}$ are the area fractions of the solid and air interfaces. It is typically assumed that $cos\theta_{v}=-1$ reflecting the idealized hydrophobic nature of the air phase. Additionally, the surface fraction parameters satisfy the relationship $f_{s}+f_{v}=1$. As $f_{s}$ approaches zero, $\theta^{*}$ tends toward 180°, indicating that the droplet essentially rests upon a cushion of air, thus significantly reducing both adhesion and frictional forces.

To optimize the hydrophobic surface treatment, we systematically investigated the effects of both heating temperature and heating duration on the static contact angle of water-based droplets. The experimental results are summarized in Figure 5b,c. Three distinct temperature conditions (60 °C, 80 °C, and 100 °C) were selected to assess the thermal curing behavior of Teflon-coated glass substrates (Excel S2). At 60 °C, the measured contact angle initially increased with extended heating time, indicating progressive enhancement of surface hydrophobicity. However, beyond a certain temporal threshold, a decline in contact angle was observed, which was due to thermal degradation or uneven polymer cross-linking. In contrast, heating at 80 °C and 100 °C led to a continuous and monotonic increase in contact angle over time. The surface treated at 80 °C exhibited a plateau in hydrophobicity after approximately 10 min. In contrast, the surface treated at 100 °C not only achieved the highest contact angle but also showed a continued upward trend without saturation within the tested time range. Based on these results, we concluded that uniform application of a Teflon coating followed by thermal curing at 100 °C for 10 min yields a hydrophobic surface with optimal wetting resistance, making it particularly well-suited for droplet-based actuation on the microfluidic chip.

### 3.5. Magnetic Droplet Manipulation

Following the validation of magnetic manipulation precision and the optimization of hydrophobic surface treatment parameters, we proceeded to assess the capability for droplet-level actuation of the platform. The electromagnetic control platform was programmed with predefined lateral and longitudinal displacement sequences using the previously optimized control parameters. As illustrated in Figure 5d, the magnetic droplet was able to traverse both horizontal and vertical trajectories smoothly, confirming the platform’s efficacy in executing directional motion with high fidelity.

To further demonstrate the platform’s applicability in bioanalytical contexts, we conducted a droplet fusion experiment to emulate a fundamental operation commonly encountered in biochemical assays. The micropipette used in this study had a nominal dispensing range of 0.1 μL to 2.5 μL. However, due to practical constraints associated with manual pipetting and surface tension effects on the hydrophobic substrate, droplets with volumes below 0.5 μL were difficult to reliably deposit and maintain on the treated glass surface. Consequently, all experiments were conducted using sample droplets with volumes ranging from 0.5 μL to 2.5 μL. The sample droplets used in this study include a 2.5 μL magnetic droplet and a 2.5 μL deionized water droplet. The magnetic droplet was prepared by vigorously mixing 0.5 μL of water-based ferrofluid EMG 900 with 2 μL of deionized water. As depicted in Figure 5e, a 2.5 μL magnetic droplet was actuated laterally toward a 2.5 μL non-magnetic aqueous droplet. Upon contact, spontaneous coalescence occurred, forming a single, merged droplet. Notably, as shown in Figure 5f, the electromagnetic platform retained full control over the merged droplet, enabling continued actuation post-fusion. Therefore, the effective operational droplet volume range is extended to approximately 0.5 μL to 5 μL (Video S1). These results underscore the platform’s potential for supporting essential microfluidic operations such as reagent mixing, sample preparation, or reaction initiation in portable diagnostic systems (Table 1).

## 4. Conclusions

In this study, we developed a programmable and portable electromagnetic microfluidic droplet manipulation platform capable of achieving robust, contactless droplet control through a low-power, coil-array-based magnetic actuation scheme, while ensuring high stability and reproducibility. Systematic characterization of magnetic field intensity, thermal output, coil activation timing, and magnet geometry revealed that driving a three-layer coil array with 180 mA for 300 ms, with 100 ms intervals, enabled stable navigation of 2.5 mm × 1 mm cylindrical magnets along complex trajectories such as “S”, “H”, and “U”, as well as the simultaneous control of up to four magnets. The inherent programmability significantly enhances the system’s scalability and operational flexibility, enabling seamless adaptation to a broad spectrum of experimental requirements. Such attributes are of critical importance for the advancement of next-generation digital microfluidic systems. Importantly, the resulting surface temperatures remained well within biocompatible limits, ensuring safe operation in bioanalytical contexts. To reduce droplet–substrate friction, the glass surface was modified using Teflon spin-coating. A curing protocol of 100 °C for 10 min produced the highest water contact angle (126°), enabling smooth magnetic droplet transport and successful on-chip droplet fusion. Together, these results underscore the potential of the proposed platform as a compact, energy-efficient, and programmable solution for droplet operations in digital microfluidics, with promising applications in point-of-care diagnostics and portable lab-on-chip systems.

## Figures and Tables

**Table 1:** The various parameters of the platform and the final achieved performance.

Parameter	Value
Driving voltage	3.5 V
Driving current	180 mA
Power consumption	0.63 W
Coil array size	16 × 16
Coil dimension	1.5 mm × 1.5 mm
Droplet volume range	0.5–5 μL
Maximum droplet velocity	0.6 cm/s
Positioning accuracy	~0.1 mm
Maximum displacement	26.25 mm
Surface temperature	<26 °C
Control method	FPGA-based
Actuation type	Hybrid magnetic
Programmability	Yes

## Data Availability

The original contributions presented in this study are included in the article/Supplementary Materials. Further inquiries can be directed to the corresponding authors.

## Supplementary Materials

The following supporting information can be downloaded at: https://www.mdpi.com/article/10.3390/bios16040196/s1, Video S1 caption: Magnetic droplet manipulation; Excel S1: deviation_torence; Excel S2: contact_angle_variation. Figure S1: 3D_structure.
