# Supplementary figures and images for: A Programmable and Portable Electromagnetic Microfluidic Platform for Droplet Manipulation
Source: Biosensors (Basel). 2026 Mar 31;16(4):196. doi: 10.3390/bios16040196 (PMC13114064; doi:10.3390/bios16040196)

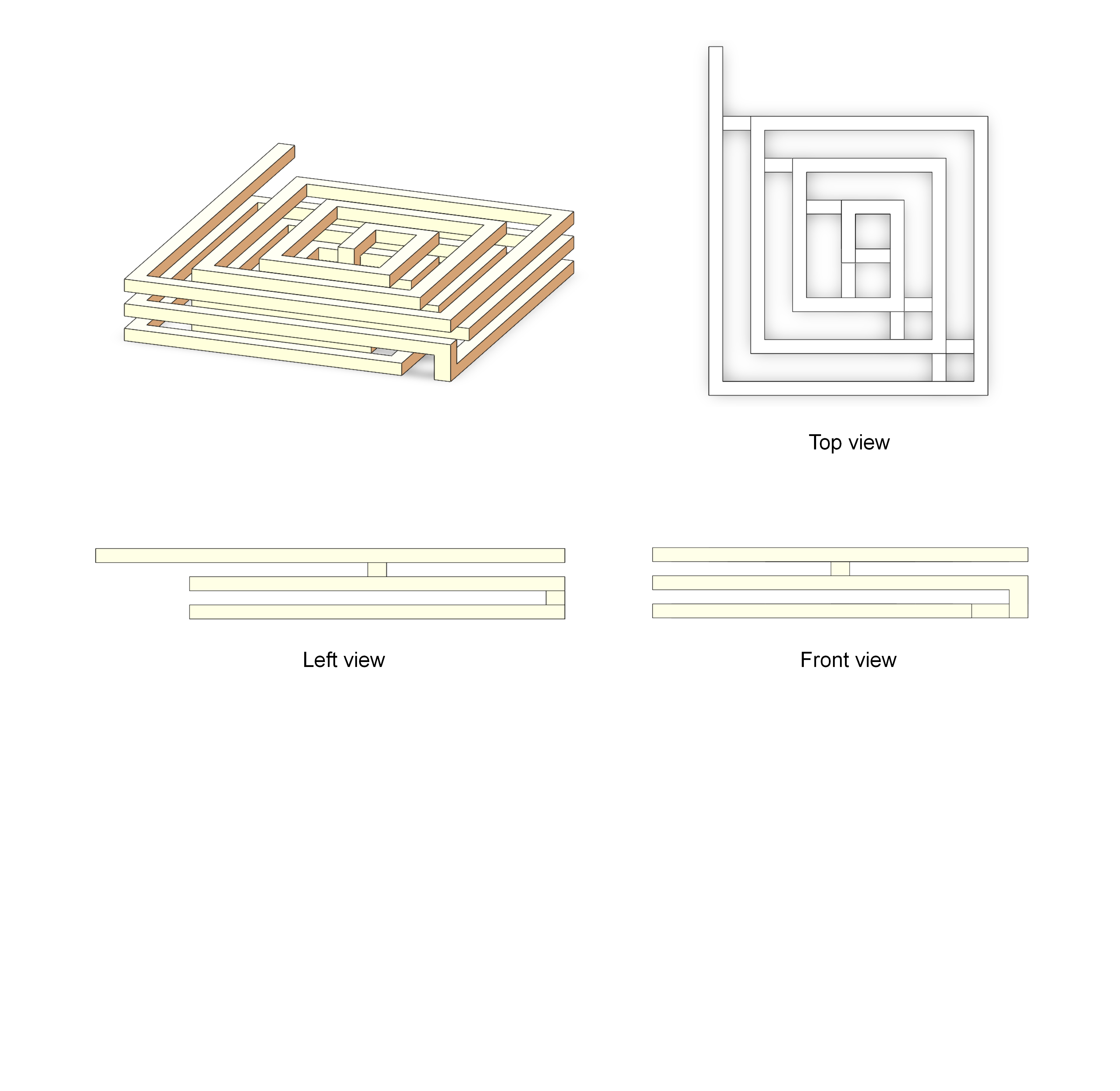

Supplement: Supplementary file 1 [file biosensors-16-00196-s001.zip › 3D_structure.png]
